# Anion Channel Inhibitor NPPB-Inhibited Fluoride Accumulation in Tea Plant (*Camellia sinensis*) Is Related to the Regulation of Ca^2+^, CaM and Depolarization of Plasma Membrane Potential

**DOI:** 10.3390/ijms17010057

**Published:** 2016-01-05

**Authors:** Xian-Chen Zhang, Hong-Jian Gao, Tian-Yuan Yang, Hong-Hong Wu, Yu-Mei Wang, Zheng-Zhu Zhang, Xiao-Chun Wan

**Affiliations:** 1 State Key Laboratory of Tea Plant Biology and Utilization, Anhui Agricultural University, Hefei 230036, China; zhangxianchen360@163.com (X.-C.Z.); hjgao@ahau.edu.cn (H.-J.G.); zzz@ahau.edu.cn (Z.-Z.Z.); 2 School of Resources and Environment, Anhui Agricultural University, Hefei 230036, China; ymwang1008@163.com; 3 College of Resources and Environmental Science, Nanjing Agricultural University, Nanjing 210095 China; yangtianyuan2008@163.com; 4 School of Land and Food, University of Tasmania, Hobart, Tasmania 7001, Australia; Honghong.Wu@utas.edu.au

**Keywords:** Ca^2+^ efflux, Ca^2+^ fluorescence, CaM, fluoride, NPPB, tea plant

## Abstract

Tea plant is known to be a hyper-accumulator of fluoride (F). Over-intake of F has been shown to have adverse effects on human health, e.g., dental fluorosis. Thus, understanding the mechanisms fluoride accumulation and developing potential approaches to decrease F uptake in tea plants might be beneficial for human health. In the present study, we found that pretreatment with the anion channel inhibitor NPPB reduced F accumulation in tea plants. Simultaneously, we observed that NPPB triggered Ca^2+^ efflux from mature zone of tea root and significantly increased relative CaM in tea roots. Besides, pretreatment with the Ca^2+^ chelator (EGTA) and CaM antagonists (CPZ and TFP) suppressed NPPB-elevated cytosolic Ca^2+^ fluorescence intensity and CaM concentration in tea roots, respectively. Interestingly, NPPB-inhibited F accumulation was found to be significantly alleviated in tea plants pretreated with either Ca^2+^ chelator (EGTA) or CaM antagonists (CPZ and TFP). In addition, NPPB significantly depolarized membrane potential transiently and we argue that the net Ca^2+^ and H^+^ efflux across the plasma membrane contributed to the restoration of membrane potential. Overall, our results suggest that regulation of Ca^2+^-CaM and plasma membrane potential depolarization are involved in NPPB-inhibited F accumulation in tea plants.

## 1. Introduction

Fluoride (F) is phytotoxic to most plants by influencing a series of basal metabolism and enzyme activities [1]; however, it can be accumulated in tea plants. Fluoride content in tea leaves ranges from hundreds to thousands of milligrams, which is 20 to 30 times higher than that of other edible plants [2]. Approximately 40% to 90% of fluoride in tea leaves can be dissolved in tea-based liquids and accumulated in the human body through tea drinking [3]. The uptake of fluoride through drinking tea with normal fluoride concentrations is somewhat considered to be safe and even beneficial to health; however, over-intake of fluoride through excessive daily consumption of brick tea causes dental and skeletal fluorosis [4,5]. As tea plants are F hyper-accumulators, understanding the underlying mechanisms beyond F accumulation is beneficial to control F accumulation in tea plants in agricultural practices. Previous study found that anion channel inhibitor A-9-C (anthracene-9-carboxylic acid) or NFA (niflumic acid) significantly blocked F^−^ net uptake (*Longjing 43*) [6]. In our lab, we reported that an anion channel inhibitor NPPB (5-nitro -2-(3-phenylpropylamino) benzoic acid) inhibited anion channels activity in tea roots and significantly reduced F accumulation in tea plants (*Fuding*) [7]. However, the possible mechanism involved in NPPB-inhibited F uptake in tea plants via anion channels was not yet investigated and still largely unknown.

Anion channels, integral membrane proteins that form aqueous pores, regulate transport of anions across the membrane [8,9]. Most anion channels are located on plant membranes, such as the plasma membrane [10,11], tonoplast [12], and mitochondria membrane [13]. Nevertheless, several lines of evidences have shown that Ca^2+^, acts as a ubiquitous intracellular signal messenger, modulating anion channels distributed at the plasma membranes in higher plants [14,15]. For example, the increase in Ca^2+^ concentration^-^activated anion channels was found in guard cells [16], pollen tubes [17], and Nicotiana tabacum [18]. In addition, ABA (abscisic acid) or pathogen-regulated Ca^2+^ oscillations activated anion channels in *Arabidopsis thaliana* guard cells and root [19,20].

Ca^2+^ signatures are decoded by several Ca^2+^ sensors such as calmodulin (CaM), calcium-dependent protein kinase (CDPK), and calcineurin B-like protein (CBL). CaM is a small acidic protein that contains four EF (elongation factor) hands, and is one of the best-characterized Ca^2+^ receptors [21]. The binding of Ca^2+^ to CaM induces a conformational change of ion channel [22,23,24,25]. Furthermore, most anion channels belong to the class of voltage-dependence, and regulate anion influx and efflux in plant root through controlling their open and closed states according to the electrochemical gradients [26,27,28]. NA (niflumic acid) induced membrane depolarization and depressed anion channel activity in maize roots, thereby regulating NO_3_^−^ and Cl^−^ efflux [29]. Besides in anion channels, modulation of membrane potential was also found to be involved in regulating other ion channels, e.g., the K^+^ channel [30]. However, the connection between Ca–CaM, anion channels, and membrane potential in F accumulation in tea plants is still obscure.

To investigate whether Ca^2+^ and CaM integrated in NPPB inhibited F accumulation in tea plants, Ca^2+^ flux, intracellular Ca^2+^ fluorescence intensity, and CaM level in tea roots were examined. Additionally, Ca^2+^ chelator EGTA (ethylene glycol tetraacetic acid), CaM antagonist CPZ (chlorpromazine hydrochloride), and TFP (trifluoperazine dihydrochloride) were also used to investigate the role of Ca^2+^ and CaM in the NPPB-inhibited F accumulation in tea plants. Further, we studied membrane potential, net H^+^ flux, and plasma membrane H^+^-ATPase activity in tea roots to investigate the possible role of regulation of membrane potential in NPPB-inhibited F accumulation in tea plants. Taken together, the present study offers some potential clues to benefit the understanding of possible regulation mechanisms beyond NPPB-inhibited F accumulation in tea plants.

## 2. Results

### 2.1. NPPB Significantly Inhibited F Accumulation in Tea Roots and Its Whole Plant

**Figure 1:**
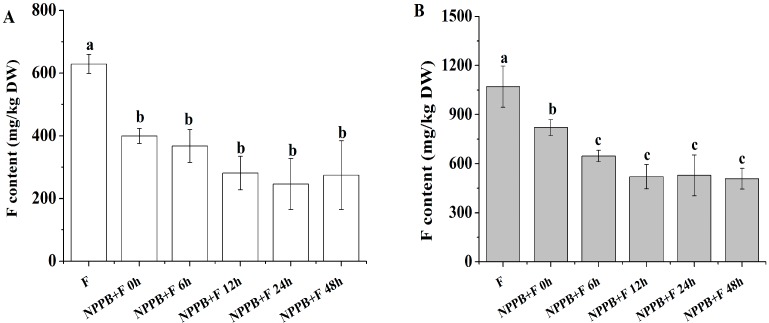
Effect of NPPB on F concentration in tea roots (**A**) and plants (**B**) with different pretreatment times. Data indicate mean ± SD (*n* = 4). Different low case numbers above the chart bars indicate the level of significance compared with the case without the addition of NPPB at *p* < 0.05.

In this study, the amounts of F accumulated in tea roots and in tea plants were 629.01 and 1070.19 mg/kg at the concentration of 200 mg/L fluoride for 1 day, respectively. Pretreatment with NPPB significantly inhibited F content by 36.52% and 23.37% as compared with the control roots and the tea plants, respectively (Figure 1).

To further estimate the timing effect of NPPB treatment on inhibition of F accumulation, the F content in tea roots and plants was monitored under different NPPB pretreatment times. Results in Figure 1A showed that F content in tea roots gradually decreased by 41.61% and 55.32% after the addition of NPPB in solution for 6 and 12 h, respectively. Meanwhile, these values were reduced by 39.56% and 51.40%, respectively in whole tea plants (Figure 1B). After 12 h treatment of NPPB, a very similar accumulation of F content was found at the level of either tea roots (Figure 1A) or whole plants (Figure 1B). Thus, all further studies were conducted at this treatment time.

### 2.2. The Changes of Net Ca^2+^ Flux and Cytosolic Ca^2+^ Intensity in Tea Roots in Response to NPPB

**Figure 2:**
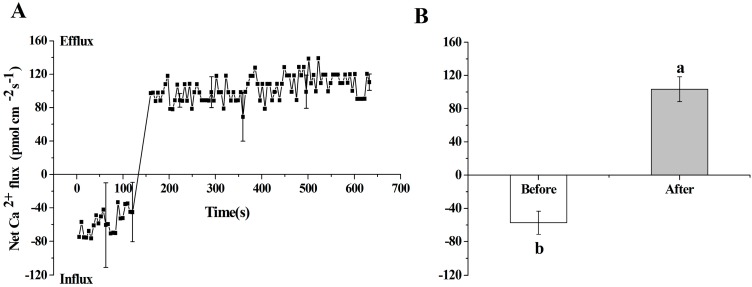
NPPB-induced Ca^2+^ flux in tea root on the mature zone cells. (**A**) The kinetics of net Ca^2+^ flux in tea root mature zone cells treated with NPPB; (**B**) Net Ca^2+^ flux at state of before (0 to 120.76 s, without 50 µM NPPB), and after (160 to 632.5 s, with NPPB). Data indicate mean ± SD (*n* = 6). Error bars indicate difference among the treatments and different low case numbers near the chart bars indicate the level of significance as compared with control at *p* < 0.05.

As mentioned in the introduction, various extracellular stimuli elicit specific calcium signatures and the production of Ca^2+^ oscillation modulates ion channel activity in plants. Therefore, the effect of NPPB on Ca^2+^ signal in tea root was investigated. By using NMT (Non-invasive Micro-test Technique), calcium flux was measured in the maturation zone of tea root under NPPB treatment. As shown in Figure 2, the influx of Ca^2+^ remained stable at a range of −72.55 to −89.26 pmol·cm^−2^·s^−1^ for 120.75 s in the absence of NPPB. The application of NPPB caused a rapid Ca^2+^ efflux at a range of 68.93 to 128.76 pmol·cm^−2^·s^−1^ between 160 and 632.5 s (Figure 2A). The mean Ca^2+^ flux value reached 103.37 pmol·cm^−2^·s^−1^, which was significantly higher by 97.69% as compared with the control (Figure 2B).

**Figure 3:**
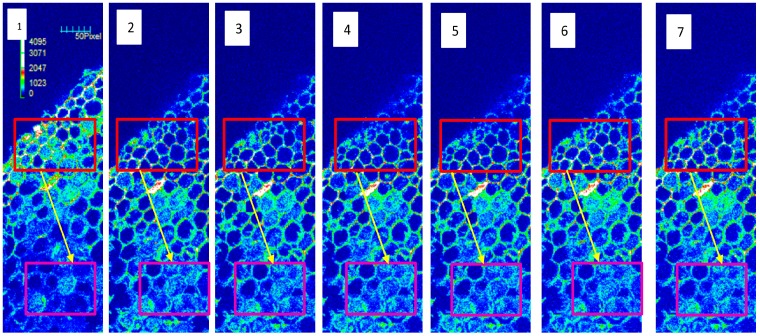
Effect of NPPB on the Ca^2+^ signal in tea root mature zone cells. 3D Ca^2+^ pseudo-color images in tea root mature zone cells in the absence of NPPB (**1**) and presence of NPPB treatment (**2**–**7**); Lanes **2** to **7** represent 3D Ca^2+^ pseudocolor images of tea roots cells at 2, 4, 6, 8, 10, and 12 min after NPPB treatment, respectively. Red represents region A; purple represents region B; arrows represent the direction of Ca^2+^ transmission.

To visualize clearly the kinetics of Ca^2+^ change induced by NPPB, a non-invasive loading method was used to load the Ca^2+^-sensitive fluorescent probe Fluo-3/AM ester into the mature zone cells of tea roots. We created a 3D-LSCM (laser scanning confocal microscope) image of tea root cells for 12 min without the addition of NPPB into the solution. The relative fluorescence intensity did not significantly change within 12 min cited from [31]. The treatment of NPPB caused different trends of calcium fluorescence intensity in two regions of maturation zone of tea roots (Figure 3). The Fluo-3 fluorescence intensity of region A decreased from 889.60 to 452.2 AU in 7 min followed with a relatively stable level from 536.4 to 594 AU after 8 min under NPPB condition. However, NPPB caused an increase in the calcium fluorescence intensity of the region B. The intracellular calcium fluorescence intensity in the mature zone cells of tea root was 371.14 AU in the absence of NPPB, but this value increased to 515.28 AU after 2 min and then kept stable from 497.85 to 539.42 AU with the addition of NPPB (Figure S1).

### 2.3. The Pretreatment of EGTA on NPPB-Induced Change of Ca^2+^ Fluorescence Intensity

**Figure 4:**
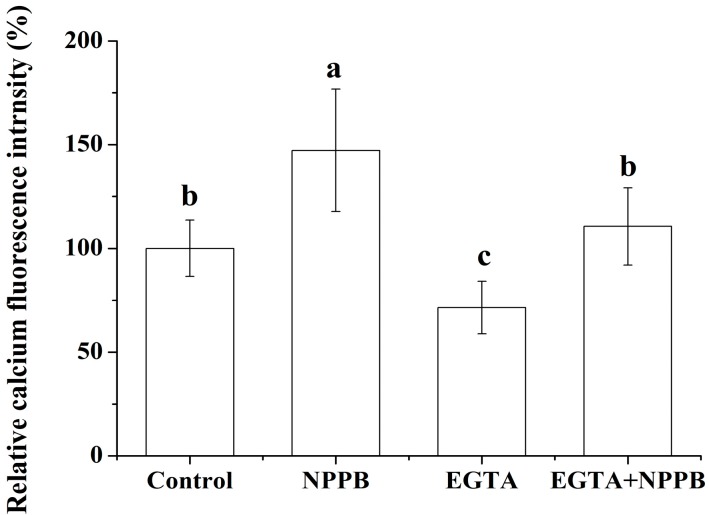
Effect of Ca^2+^ chelator (EGTA) on cytosolic Ca^2+^ fluorescence intensity in NPPB-treated tea roots. Control, NPPB and EGTA indicate distilled water, 50 µM NPPB, and 1 mM EGTA for 0.5 h respectively; EGTA + NPPB refers to tea roots were placed in 1 mM EGTA for 0.25 h, then subjected to a direct 50 µM NPPB for 0.5 h; Ca^2+^ fluorescence was determined after control, NPPB, EGTA and EGTA + NPPB treatment in tea lateral roots. Data indicate mean ± SD (*n* = 4). Error bars indicate difference among the treatments and different low case numbers near the chart bars indicate the level of significance as compared with control at *p* < 0.05.

To further analyze the role of Ca^2+^ in the inhibition of F accumulation in tea plants, EGTA was used in the following experiment. In contrast to the lowest level of intracellular Ca^2+^ fluorescence intensity in lateral root treated with EGTA, the highest one was found in NPPB treatment in comparison with control treatment. NPPB-elevated (*p* < 0.05) intracellular Ca^2+^ fluorescence intensity in lateral root was significantly suppressed to a level similar to the control by EGTA pretreatment (Figure 4).

### 2.4. Effects of Ca^2+^ Chelator and CaM Antagonists on NPPB-Induced CaM Content

Transient Ca^2+^ elevations are sensed by Ca^2+^ receptors and CaM is one of the best-characterized calcium sensors in plants. Thus CaM level in tea roots exposed to NPPB was investigated. The CaM concentration in tea roots in the control treatment (distilled water) stayed constant throughout the 4 h of the experiment, ranging from 77.62 to 89.27 ng·g^−1^. However, NPPB-stimulated tea roots caused a significant (*p* < 0.05) fluctuation of the CaM content at 2 h; it was higher by 37.94% as compared with the controls. Afterwards, the value returned to a level similar to that of control at 4 h (Figure S2).

**Figure 5:**
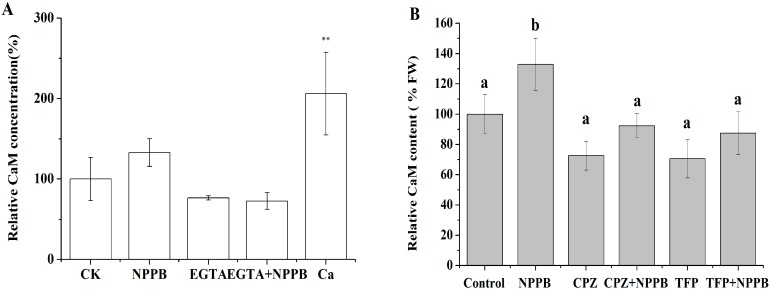
Effect of Ca^2+^ chelator (EGTA) and CaM antagonists (CPZ and TFP) pre-treatment on CaM levels in NPPB-treated tea roots, respectively. (**A**) Control, NPPB, EGTA and Ca^2+^ indicate the application of distilled water, 50 µM NPPB, 1 mM EGTA and 1 mM Ca^2+^ for 2 h, respectively; EGTA + NPPB refers to tea roots were placed in 1 mM EGTA for 0.5 h, then directly subjected to 50 µM NPPB for 2 h; (**B**) Control, NPPB and CPZ, TFP indicate distilled water, 50 µM NPPB and 50 µM CPZ or TFP for 2 h, respectively; CPZ + NPPB and TFP + NPPB refer to tea roots were placed in 50 µM CPZ and TFP for 0.5 h respectively, then subjected to a direct 50 µM NPPB for 2 h. CaM level was detected after control, NPPB and CPZ, TFP, CPZ + NPPB and TFP + NPPB treatment in tea roots. Data indicate mean ± SD (*n* = 4). Error bars indicate differences among the treatments and different low case numbers near the chart bars indicate the level of significance as compared with control at *p* < 0.05; Note: The data for Ca^2+^ treatment was adapted from [31].

To further illustrate the relationship between Ca^2+^ and CaM, the Ca^2+^ chelator EGTA was applied (Figure 5A). EGTA treatment significantly reduced the CaM concentration by 24.40% as compared with the controls at 2 h, in contrast to significant higher relative CaM content in tea roots treated with NPPB than control. In addition, after tea roots were placed in 1 mM EGTA for 0.5 h then subjected to NPPB for 2 h, similar results of CaM protein level were found between treatments of EGTA and EGTA + NPPB. These results indicate that CaM accumulation in tea roots induced by NPPB was dependent on Ca^2+^ in tea roots. Not surprisingly, about one fold higher relative CaM content was found in tea roots supplied with Ca^2+^ than control. Significant reduced relative CaM content than control was found in tea roots treated with CaM(calmodulin) antagonists CPZ (chlorpromazine hydrochloride)and TFP (trifluoperazine dihydrochloride), whereas these treatments were not sufficient to reduce relative CaM content in tea roots when co-treated with NPPB (Figure 5B).

### 2.5. Endogenous Ca^2+^ and CaM Involved in NPPB-Inhibited F Accumulation in Tea Plants

**Figure 6:**
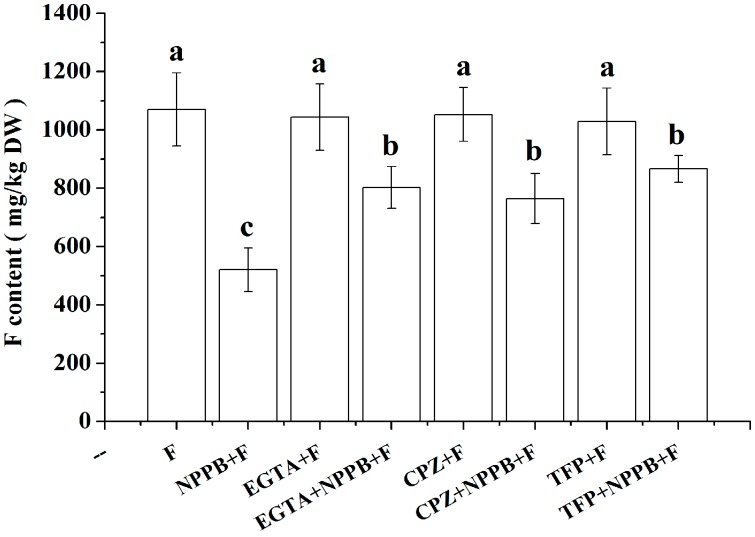
Effect of EGTA, CPZ and TFP pre-treatment on relative F accumulation in NPPB-treated tea plants. NPPB + F indicates tea roots were applied with 50 µM NPPB for 12 h and then followed with 10.5 mM F for 1 day; EGTA + F, CPZ + F, and TFP + F indicate that 6 h pretreament with 1 mM EGTA, 50 µM CPZ and 50 µM TFP and then treated with F treatment; EGTA + NPPB + F, CPZ + NPPB + F, and TFP + NPPB + F indicate that 6 h pretreament with 1 mM EGTA, 50 µM CPZ and 50 µM TFP were applied to the tea roots under NPPB + F treatment, respectively. Data indicate mean ± SD (*n* = 4). The error bars indicate difference among the treatments and different low case numbers near the chart bars indicate the level of significant difference compared with control at *p* < 0.05.

The above results indicate that NPPB stimulated internal Ca^2+^ and CaM level in tea roots, and that the Ca^2+^ chelator EGTA or CaM antagonist CPZ and TFP decreased NPPB induced Ca^2+^ and CaM levels. Therefore, the effect of EGTA, CPZ, and TFP was examined to test the role of Ca^2+^ and CaM in the NPBB-inhibited F accumulation in tea plants. Firstly, no significant inhibition effect of EGTA, CPZ and TFP on F content in tea plants was found. However, we found that NPPB-inhibited F accumulation in tea roots was significantly alleviated using Ca^2+^ chelator EGTA. Similarly, the pretreatment of CaM antagonist CPZ and TFP remarkably impaired NPPB-inhibited F accumulation in tea plants (Figure 6).

### 2.6. NPPB Depolarized Membrane Potential and Stimulated the Net H^+^ Effluxes in the Maturation Zone of Tea Roots

**Figure 7:**
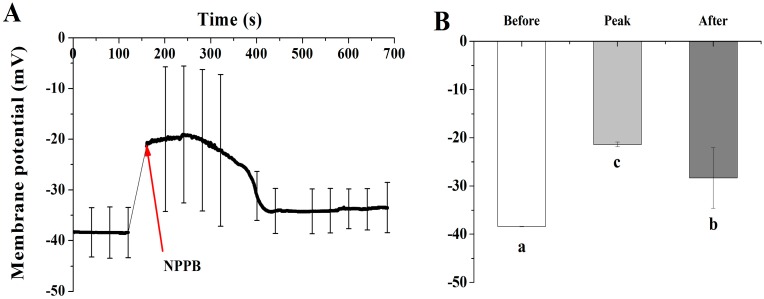
Effect of NPPB on the membrane potential in tea root mature zone cells. (**A**) The kinetics of plasma membrane potential in tea root mature zone cells treated with NPPB; (**B**) Plasma membrane potential at state of before (0 to 120.75 s, without 50 µM NPPB), peak (160 to 300.96 s, with NPPB), and after (120.75 s, with NPPB). Data indicate mean ± SD (*n* = 8). Error bars indicate the difference among the treatments and different low case numbers near the chart bars indicate the level of significant difference compared with control at *p* < 0.05.

**Figure 8:**
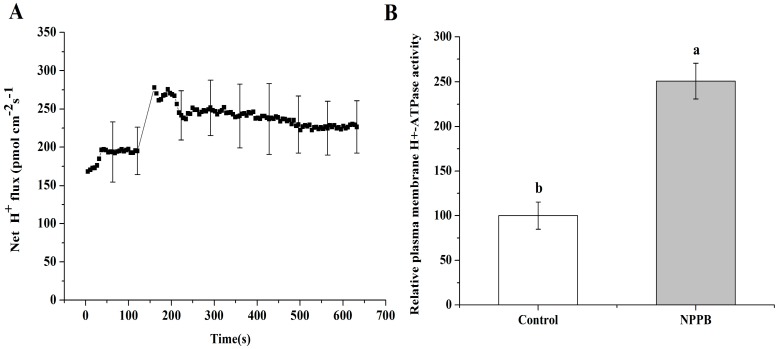
Influence of NPPB on the H^+^ flux and plasma membrane H^+^-ATPase activity in tea roots. (**A**) The kinetics of net H^+^ flux in tea root mature zone cells treated with NPPB. Data indicate mean ± SD (*n* = 6). Error bars indicate differences among the treatments; (**B**) Control and NPPB indicate that tea roots were treated with distilled water and 50 µM NPPB for 12 h, respectively; Data indicate mean ± SD (*n* = 4). Error bars indicate differences among the treatments and the letters a, b near the chart bars indicate the level of significance as compared with control at *p* < 0.05.

In plant cells, most of anion channels are related to voltage dependence. Thus the regulation of anion channels’ activity in tea roots under stimuli might be related to modulation of its plasma membrane potential. In our study, NPPB treatment caused the instant depolarization of plasma membrane potential (Figure 7A). Then the extent of the membrane potential depolarization gradually diminished from 300.96 to 684.64 s (Figure 7A,B). Not surprisingly, increased net H^+^ efflux was found in tea roots treated with NPPB (Figure 8A). Consistently, plasma membrane H^+^-ATPase activity was significantly activated by 149.44% in tea root under NPPB condition than control (Figure 8B).

## 3. Discussion

Previous results from our laboratory have shown that the anion channel inhibitor NPPB significantly decreased F absorption, suggesting that anion channels are involved in F accumulation in tea plants [6]. However, the components in regulation of NPPB inhibited F accumulation in tea plants are still obscure.

### 3.1. Ca^2+^ and CaM Are Involved in NPPB-Inhibited F Accumulation in Tea Plants

Ca^2+^ is a well-known important second messenger, and a wide range of stimuli trigger a change of the intracellular Ca^2+^ concentration [32,33,34]. In the present work, a net Ca^2+^ efflux (Figure 2) and a change of cytosolic Ca^2+^ intensity (Figure 3) was seen in tea root in response to the anion channel inhibitor, NPPB. Ion channel inhibitor-activated Ca^2+^ signals were also reported in other plants. Chen *et al.* [35] found that the addition of Ca^2+^ channel inhibitor La^3+^ obviously elevated Ca^2+^ concentration in the cytoplasm in *Picea meyeri* pollen tube. GdCl_3_, a nonspecific cationic channel blocker, induced Ca^2+^ influx and increased Ca^2+^ concentration in the cytoplasm in the gamete cells of maize [36]. Similar results were also found in plants, e.g., rice root [37] and populus euphratica cells [38] under other abiotic stress. Further, EGTA decreased NPPB-increased Ca^2+^ fluorescence intensity in the lateral root (Figure 4) and impaired NPPB-inhibited F accumulation in tea plants (Figure 6), which suggests that intracellular Ca^2+^ change was related to NPPB inhibited F accumulation in tea plants. Roberts *et al.* [39] reported that an anion channel inhibitor niflumic acid depressed anion channel and modulated NO_3_^−^, Cl^−^, and I^−^ uptake in maize root stele via Ca^2+^ signal regulation. Similar result of involvement of Ca^2+^ in regulation anion channels was also found in other plant species, e.g., *Chara corallina* [40].

CaM is a primary calcium sensor in most of eukaryotes. It binds calcium and regulates the activities of a wide range of effect proteins in response to calcium signals [41]. We found that CaM accumulation induced by NPPB is dependent on Ca^2+^ in tea roots in 4 h (Figure 6A) and the inhibition of NPPB on F accumulation in tea plants were significantly alleviated by the presence of either Ca^2+^ chelator EGTA or the CaM antagonists CPZ and TFP (Figure 6B), suggesting that both Ca^2+^ and CaM are involved in NPPB inhibited F accumulation in tea plants. Similarly, results showed that NaCl stress-regulated Na^+^ and K^+^ uptake was associated with CaM in barley roots [42]. However, it should be noted here that CaM antagonists TFP and CPZ can not only inhibit CaM but also impair the function of CBL and CDPK [43]. Several lines of evidences showed that CDPK and CBL involved in ion uptake are well known. For example, Pei *et al.* reported that CDPK activated chloride channels in *Vicia faba* guard cell vacuoles, which enabled Cl^−^ entry into vacuoles [44]. Cheong *et al.* found that CBL1 and CBL9 are involved in regulating K uptake and transport in *Arabidopsis* root under low K condition [45]. Thus, it is possible that Ca^2+^ sensors CDPK or CBL might also interact with components involved in NPPB-inhibited F accumulation in tea plants. To better understand the mechanisms behind F accumulation in tea plants, CDPK and CBL might be interesting targets in future studies.

### 3.2. A Possible Link between Regulation of Ca-CaM and Plasma Membrane Potential in NPPB-Inhibited F Accumulation in Tea Plants

NPPB inhibited anion channels and significantly reduced F accumulation in tea plants. As described above, the regulation of anion channel activity in tea root under stimuli might also be related to regulation of its plasma membrane potential. In the present study, we found that NPPB caused a rapid depolarization of membrane potential and then the extent was gradually impaired (Figure 7). In addition, NPPB significantly promoted Ca^2+^ (Figure 2A) and H^+^ efflux (Figure 8A) in tea root, which in turn contributed to alleviate membrane potential depolarization. Similarly, Shabala *et al.* [46] and Cuin *et al.* [47] reported that NaCl rapidly depolarized the plasma membrane and resulted in net Ca^2+^ and H^+^ efflux in barely roots, which weakened the extent of membrane potential depolarization. Also, a restoration of depolarized plasma membrane potential and increased plasma membrane H^+^-ATPase activity were found in barely roots under salt stress [48]. Taken together, a possible link between regulation of plasma membrane potential and Ca–CaM in NPPB inhibited F accumulation in tea plants is suggested. Buchanan *et al.* [28] reported that environmental stimuli increased the concentration of cytosolic free Ca^2+^ and evoked a rapid depolarization of the membrane potential, which activated K^+^ channel in Arabidopsis root. Similar results were also found in NaCl-stimulated Populus euphratica cells [49]. Lamottea *et al.* showed evidence that NO mediated free cytosolic Ca^2+^ concentration and activated plasma membrane Ca^2+^ channels by inducing rapid membrane potential depolarization in Nicotiana plumbaginifolia (It is correct)cells under abiotic stress [50]. Additionally, binding with Ca^2+^ and calmodulin was shown to be induce the dimerization of K^+^ channel domains, leading to channel gating and then directly coupling cytosolic Ca^2+^ change and altered plasma membrane potential [22]. Thus, in combination with our results, one possible mechanism for NPPB-inhibited F accumulation in tea plants can be proposed: NPPB triggers membrane potential depolarization, generates Ca^2+^ signal, and then rearranges anion channel conformation through interaction with increased CaM activity in tea root cells; thus, resulting in NPPB-inhibited F accumulation in tea plants. However, whether the possible link between regulation of Ca–CaM and NPPB evoked depolarization of plasma membrane potential in tea root is specific or not remains unknown. Further investigation of the regulation network between cytosolic Ca^2+^, CaM, plasma membrane potential, and anion channel activity might be beneficial to understanding the mechanism beyond anion channel inhibitor/blocker e.g., NPPB-inhibited F accumulation in tea plants. Furthermore, it is generally known that NPPB is a Cl^−^ channel inhibitor and Cl^−^ is the predominant permeating anion species in all organisms, and thus anion channels are often referred to as Cl^−^ channels [51]. However, the identity of channels in mediating F uptake in tea plants is still largely unknown. In our study, we found that NPPB (known as Cl^−^ channel inhibitor) significantly inhibited F accumulation in tea plants. Whether F uptake in tea roots was mediated by Cl^−^ anion channels, e.g., F^−^/Cl^−^ co-transport or by its specific F^−^ uptake channel still needs to be clarified in future study.

## 4. Materials and Methods

### 4.1. Cultivation of Tea Plants

Tea seeds of the Fuding variety (*Camellia sinensis cv. Fuding-dabaicha*) were obtained from the tea garden of Anhui Agricultural University located in Hefei, Anhui province of China. These seeds were first immersed in water for 2 days. Healthy tea seeds were placed on clean quartz sand (particle size—0.3 cm), and then transferred into an artificial climate chamber for one month with a day length of 12 h per day, temperature of 22 ± 1°C, irradiance of 27 µmol·m^−2^·s^−1^, and relative humidity of 45% to 50% after germination. Germinated seeds (root length of 2–5 cm) with similar growth conditions were used to determine the net Ca^2+^/H^+^ flux, intracellular Ca^2+^ concentration, CaM level, and membrane potential in tea roots.

Tea plants (grown for 3 months) at the same growth conditions (three to five leaves) were washed with deionized water, and then transferred to ventilated plastic basins (50 × 30 cm) containing 25% strength of advanced Kimura nutrient solution (NH_4_NO_3_ 114 mg·L^−1^, KH_2_PO_4_ 13.6 mg·L^−1^, and KCl 38.69 mg·L^−1^; pH 5.00 to 5.50). After growing more lateral roots, the tea plants were left in the nutrient solution for one week before treatment. Water in plastic basins was renewed every 2 to 3 days before the experiment. Tea plants were used to measure plasma membrane H^+^-ATPase activity in tea roots and F content in tea plants [31].

### 4.2. NPPB Treatment

A group of 3 or 4 tea plants (20 to 25 cm high, 5 mm stem OD) at the same growth stage (3 to 5 leaves) were rinsed in deionized water to remove the excess absorbed solutions on the roots, and then dried with filter paper. The tea plants were transferred to 250 mL glass bottles containing either distilled water for the controls or 50 µM NPPB (dissolved in DMSO). The bottles were covered with absorbent cotton and wrapped with black adhesive tape to enable efficient tea root growth. Then, tea plants were exposed to NPPB solutions for 0 h (the inhibitors plus F directly for 1 day without the pretreatment with inhibitors), 6, 12, 24 and 48 h. Subsequently, they were transplanted into 10.5 mM (200 mg·L^−1^) F (NaF) solution (containing 25% strength of advanced Kimura nutrient solution) for 1 day.

### 4.3. Determination of F

Fluoride concentrations were measured as described by Zhang *et al.* [6]. Briefly, tea plants were separated into roots, stems, and leaves, respectively. They were washed with distilled water and then dried at 80 °C. Fluoride concentration was determined by a fluoride ion-measuring instrument (9609 BNWP fluoride ion selective electrode). Samples were accurately weighed, placed in a 50 mL centrifugal tube with 30 mL of distilled water, and extracted at 100 °C for 30 min in water baths. The extraction mixture was cooled to room temperature, and then centrifuged for 10 min at 1700× *g* to separate the supernatant. Fifteen mL of extracts and 15.0 mL of total ionic strength adjustment buffer (TISAB) were completely mixed in a 50 mL polyethylene beaker with a clean glass bar. The fluoride ionic electrode was immersed into the solution, and the meter reading was recorded as soon as the reading was stable. The treatment time of NPPB that caused the minimum F accumulation in tea plants was used for further experiments.

### 4.4. Flux Measurement of Ca^2+^ and H^+^

The net fluxes of Ca^2+^ and H^+^ were measured by using non-invasive micro-test technique at the Younger USA (Xuyue Beijing) NMT Service Center. The concentration of Ca^2+^ and H^+^ concentration gradients were positioned vertically 400 μm above the cell for 3 to 5 min to record the background signals and measured by moving the electrode repeatedly between two positions (5 and 35 µm). During the process, the tea root was analyzed in the measuring solution containing 0.02 mM CaCl_2_, 50 mM sorbitol, 0.3 mM MES, and pH 6.0 for 2 min. The steady Ca^2+^/H^+^ fluxes were record for 4–5 min before the application of NPPB. Subsequently, 50 µM NPPB was immediately added to the measuring solution and each sample were tested. The microelectrodes were positioned at 2 μm away from the tea root via the computer-controlled NMT system. The net Ca^2+^ and H^+^ flux was calculated by Fick’s law of diffusion:

(1) *J* = −D(d*c*/d*x*)

where *J* indicates the ion flux in the *x* direction, d*c*/d*x* is the calcium ion concentration gradient and D is the ion diffusion constant in a particular medium [52]_._

### 4.5. Measurement of Cytosolic Ca^2+^ Intensity

The cytosolic Ca^2+^ was illustrated by using Ca^2+^-sensitive fluorescent dye Fluo-3/AM ester purchased from Molecular Probes (Eugene, OR, USA) according to the method described in Zhang *et al.* [53]. The mature zone segment isolated from tea root was incubated in a solution containing 40 µM Fluo-3/AM, 50 mM sorbitol, and 0.2 mM CaCl_2_ at 4 °C for 2 h in the dark. The Fluo-3/AM ester (50 µg) was dissolved in DMSO (dimethylsulfoxide). The final DMSO concentration in the incubation solution was approximately 1% (*v*/*v*). After incubation at 4 °C for 2h, the roots were then incubated in the 0.2 mM CaCl_2_ solution for 2 h at room temperature in the dark. The mature zone segment of tea root which was covered with the cover-slip and mounted in the chamber treated with 50 µM NPPB, and the pictures were taken by scanning every 1 min with a three dimensional XY-T project step over a period of 12 min in the bathing solution. Finally, fluorescence emission from the mature zone of tea root was detected, and the wavelength of excitation light and emission was 488 and 515 nm, respectively. The recorded fluorescence intensity was the average value obtained by scanning the cells of a specific area.

### 4.6. Plasma Membrane H^+^-ATPase Assay

About three and a half grams of fresh tea roots (a group of three or four tea plants) were sampled, rinsed with distilled water, and homogenized in a mortar with ice in 18 mL of buffer solution. This buffer contained 25 mM Hepes–Tris (pH 7.6), 50 mM Mannitol, 3 mM EGTA, 3 mM EDTA, 250 mM KCl, 2 mM PMSF, 1% PVP, 0.1% BSA, and 2 mM DTT. The homogenate was filtered through four layers of cheesecloth filter and centrifuged at 10,000× *g* for 10 min. Afterwards it was centrifuged at 50,000× *g* for 45 min to obtain a microsomal fraction. Plasma membranes were isolated from the microsomal fraction by partitioning at 6.2% Dexrean T-500 and 6.2% PEG 3350 in an aqueous polymer two-phase system as described by Chen *et al.* [48]. To examine the purity of plasma membrane, the enzyme activity was reduced over 75% by vanadate, and inhibited less than 10% by nitrate and azide. These results demonstrated that the purity of plasma membranes isolated from the microsomal fraction can be used for further experiments. The plasma membrane H^+^-ATPase hydrolysis assays were performed by measuring the release of phosphate [54].

### 4.7. CaM Extraction and Analysis

Tea roots were treated with 50 µM NPPB for 0, 0.5, 1, 2 and 4 h. At the end of each experiment, the tea roots were weighted and grounded in buffer solution (50 mM Tris–HCl (pH 8.0), 1 mM EGTA, 0.5 mM PMSF (phenylmethylsulfonyl fluoride), 20 mM NaHSO_4_, and 0.15 mM NaCl) at about 1:3 (weight/volume) at 4 °C. The homogenate was kept at 90 °C to 95 °C for 3 min and then the centrifuged at 20,000× *g* for 20 min at 4 °C. The supernatants were used to determine CaM content by ELISA according to the method described in Sun *et al.* [55]. The antibody used for ELISA was purchased from Promega (Madison, WI, USA) [33]. Protein content of tea roots was determined using the method of Bradford [56] with bovine serum albumin (BSA) as standard.

### 4.8. Membrane Potential Measurements

The membrane potential was measured at the Younger USA NMT Service Center via NMT and the iFluxes 1.0 Software. The membrane potential microelectrodes were provided by the NMT Service Center and made prior to each test to ensure the best performance. Briefly, glass microelectrodes were backfilled with 3 mM KCl to ca. 1 cm in length. An Ag/AgCl wire electrode holder was inserted at the back of the electrode to make electrical contact with the electrolyte solution. The YG003-Y05, provided by the NMT Service Center was used as the reference electrode. After cell penetration, the membrane potential from the mature zone of tea roots was recorded for 2 min. Subsequently, 50 µM NPPB was added to each treatment, and each sample was measured for at least 10 min. At least eight individual roots (*n* ≥ 8) were used for membrane potential measurements [48].

### 4.9. Data Analysis

The F content, intracellular Ca^2+^ fluorescence intensity, net Ca^2+^/H^+^ flux, CaM protein concentration, and plasma membrane H^+^-ATPase activity were analyzed using Origin Pro 8.5 and SPSS. Significant differences were analyzed by one-way Tukey’s multiple range tests and regarded as statistically significant at *p* < 0.05.

## Supplementary Materials

Supplementary materials can be found at http://www.mdpi.com/1422-0067/17/1/57/s1.

## Abbreviations

ABA: abscisic acid; CaM:calmodulin; CPZ:chlorpromazine hydrochloride; EF: elogation factor; LSCM: laser scanning confocal microscope; NA: niflumic acid; NMT: non-invasive Micro-test Technique; TFP: trifluoperazine dihydrochloride.
