# Supplementary material for: Anion Channel Inhibitor NPPB-Inhibited Fluoride Accumulation in Tea Plant (Camellia sinensis) Is Related to the Regulation of Ca2+, CaM and Depolarization of Plasma Membrane Potential
Source: Int J Mol Sci. 2016 Jan 5;17(1):57. doi: 10.3390/ijms17010057 (PMC4730302; doi:10.3390/ijms17010057)
Supplement: Supplementary file 1 [file ijms-17-00057-s001.pdf]

Supplementary Materials: Anion Channel Inhibitor NPPB-Inhibited Fluoride Accumulation in Tea Plant (*Camellia sinensis*) Is Related to the Regulation of Ca^{2+}, CaM and Depolarization of Plasma Membrane Potential

Xian-Chen Zhang, Hong-Jian Gao, Tian-Yuan Yang, Hong-Hong Wu, Yu-Mei Wang, Zheng-Zhu Zhang and Xiao-Chun Wan

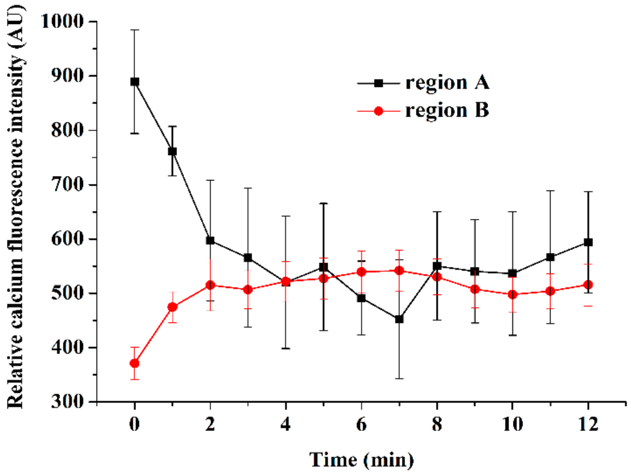

Figure S1. Kinetics of intracellular Ca^{2+} in tea root maturation zone cells during NPPB treatment. Data indicate mean ± SD (n = 3). Error bars indicate difference.

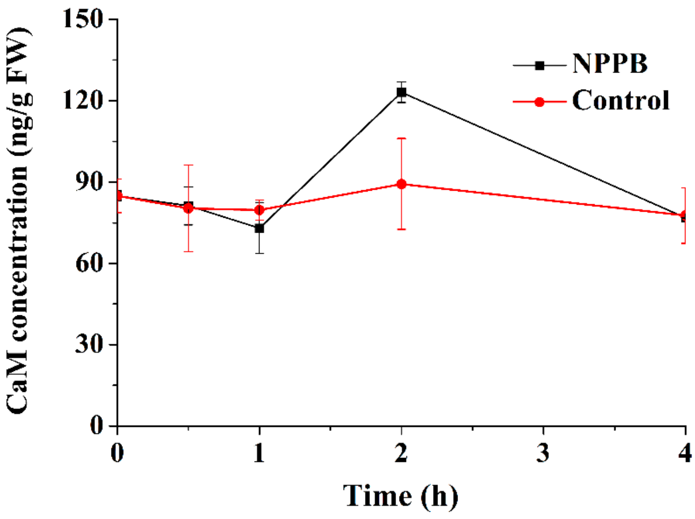

Figure S2. Effect of NPPB on CaM content of tea roots. Data indicate mean ± SD (n = 4). The error bars indicate differences among the treatments. Note: data for control treatment was adapted from [31].
